# dMyc is required in retinal progenitors to prevent JNK-mediated retinal glial activation

**DOI:** 10.1371/journal.pgen.1006647

**Published:** 2017-03-07

**Authors:** Lígia Tavares, Andreia Correia, Marília A. Santos, João B. Relvas, Paulo S. Pereira

**Affiliations:** 1 i3S –Instituto de Investigação e Inovação em Saúde, Universidade do Porto, Porto, Portugal; 2 IBMC–Instituto de Biologia Molecular e Celular, Universidade do Porto, Porto, Portugal; New York University, UNITED STATES

## Abstract

In the nervous system, glial cells provide crucial insulation and trophic support to neurons and are important for neuronal survival. In reaction to a wide variety of insults, glial cells respond with changes in cell morphology and metabolism to allow repair. Additionally, these cells can acquire migratory and proliferative potential. In particular, after axonal damage or pruning the clearance of axonal debris by glial cells is key for a healthy nervous system. Thus, bidirectional neuron-glial interactions are crucial in development, but little is known about the cellular sensors and signalling pathways involved. In here, we show that decreased cellular fitness in retinal progenitors caused by reduced *Drosophila* Myc expression triggers non cell-autonomous activation of retinal glia proliferation and overmigration. Glia migration occurs beyond its normal limit near the boundary between differentiated photoreceptors and precursor cells, extending into the progenitor domain. This overmigration is stimulated by JNK activation (and the function of its target Mmp1), while proliferative responses are mediated by Dpp/TGF-β signalling activation.

## Author summary

For a functional nervous system, neurons transmit information from cell to cell while glial cells provide crucial insulation and trophic support to neurons, which is important for neuronal survival. Glial cells are one of the most plastic cell types being able to adapt and respond to changing environmental stimuli. In this work we inhibit the function of the growth regulator dMyc in *Drosophila* retinal primordium, the eye imaginal discs. Glial cell numbers and migration pattern to the eye disc are tightly controlled but in dMyc-depleted retinas the glial cells overcome their normal barriers and overmigrate into the eye progenitors domain. We show evidence that this process is mediated by JNK activation in the presence of metalloproteinases. We discuss the biological role of overmigrating glia in tissue regeneration and/or confinement of the damaged area.

## Introduction

The nervous system is formed by neurons, which transmit information from cell to cell, and by glia, which supports and maintains a healthy and functional neuronal network (reviewed in [1]). A key function of glial cells is to provide insulation, trophic and survival support to neurons [2]. Furthermore, myelinating glia (or wrapping glia in invertebrates) can sculpt the structural and electrical properties of axons by controlling their diameter [3,4], or the spacing and clustering of ion channels at nodes and paranodes [1]. In addition, during development about 50% of neurons undergo programmed cell death (PCD) while others require axonal, dendritic, or synaptic pruning. Clearance of apoptotic corpses and engulfment of pruned parts is mediated by microglia and astrocytes in vertebrates [1,5–9] and by various types of glia in *Drosophila* [10–17].

Neurons and glia interact in a bidirectional manner as distinct neuronal signals can regulate proliferation, survival, and differentiation of glial cells [18–22]. Despite the existence of some differences, similar mechanisms are involved in both invertebrates and vertebrates to match the axon surface area requiring wrapping and the number of wrapping glial cells [23]. This is well demonstrated by the sequential increase in retinal glia number to match the differentiating photoreceptors in the *Drosophila* eye [24] and the increase of glial cell size, through polyploidization, to match the increase of neuronal mass in the growing brain [25]. Furthermore, axonal neuregulin controls the proliferation and the survival of oligodendrocytes and Schwann cells in mammals [21,26–29] and longitudinal glia in *Drosophila* [23,30]. Other factors such as PVF/PDGF and TGF-α have also been shown to exert both a gliatrophic and gliatropic function in glia [30–32]. For those functions to be accomplished, glial cells have to be extremely plastic in order to quickly respond to neuronal changes.

Despite the cumulative knowledge on the role of glia during axonal development and maintenance it is unknown if glia has the ability to recognise and respond to the microenvironment fitness changes in the context of development. Myc is a highly conserved helix-loop-helix leucine zipper transcription factor which mutations affect the ‘‘cellular fitness” at the level of cell growth and proliferation [33–38]. The conserved MYC functions include control of cell growth, energy production (glycolysis, glutaminolysis and mitochondrial biogenesis), anabolic metabolism (synthesis of amino acids, nucleotides and lipids) and DNA replication [37,38]. At the mechanistic level, MYC binds and regulates a large subset of genes that control ribosome biogenesis [39,40], translation [41,42] and metabolism [39,40,43]. In fact, the ability of Myc to stimulate ribosome biogenesis is crucial both in development and oncogenesis [44]. This happens in part through the regulation of Polymerase I [45,46] and the RNA exonuclease Viriato (Nol12 in vertebrates) [47,48]. In this work, we have impaired retinal progenitors fitness in *Drosophila* eye imaginal disc by *Drosophila Myc* (*dMyc*) loss-of-function and found that it induces glial cell proliferation and overmigration. Myc acts in eye disc progenitors to prevent JNK activation, which is otherwise sufficient to induce matrix metalloproteinase 1 (Mmp1) expression and trigger glia overmigration.

## Results

### dMyc is required in eye progenitors to block glia overmigration

**Fig 1:**
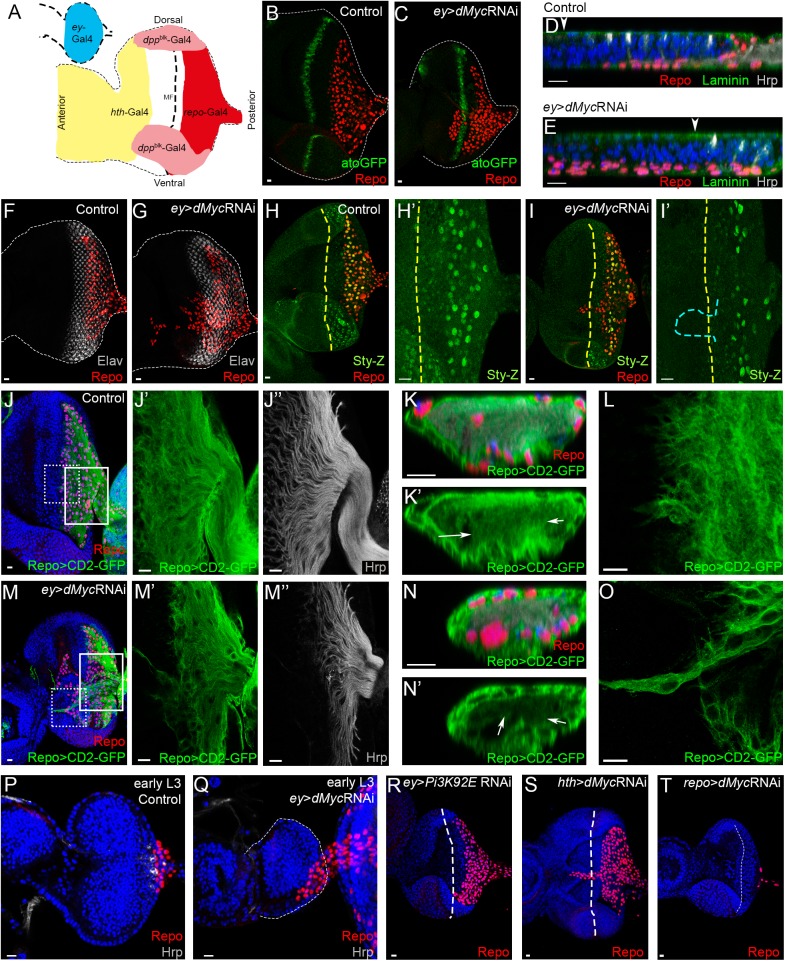
dMyc knockdown in the eye imaginal disc induces glia overmigration. (A) Schematic of the L2 (top left) and L3 (bottom) eye imaginal disc with color-coded expression domains of the Gal4 drivers used in this work. Red: *repo*-Gal4; Blue: *ey*-Gal4; Pink: *dppblk*-Gal4; Yellow: *hth*-Gal4. A dashed line represents the Morphogenetic Furrow (MF). (B–C) Atonal expression assessed by the reporter *ato-*GFP in control (B) and *ey*>*dMyc* RNAi (C). (D–E) Transverse view of the eye imaginal disc showing glia nuclei (red) and photoreceptor axons (grey) in Control (D) and *ey*>*dMyc* RNAi (E). An arrowhead indicates MF. (F–G) Photoreceptor cells stained with Elav (neuronal marker) in control (F) and *ey*>*dMyc* RNAi (G). (H–I) Wrapping glial cells are labelled with β-galactosidase to detect *sprouty*-LacZ (Spy-Z) (green) in control (H) and *ey*>*dMyc* RNAi (I). Cyan dashed line represents the glia overmigration position. (J–O) Glial cell membranes were detected with *repo*LexA-LexAopCD2-GFP (green) in control (J–L) and *ey*>*dMyc* RNAi (M–O). J’, J”, M’ and M” are magnifications of the white inset shown in panel J and M respectively. K and N correspond to transversal section of the optic stalk where wrapped axons are visible. Arrows point towards region of wrapping glia. L and O are magnifications of the dashed inset shown in panel J and M, respectively, showing glia morphology at the edge of migration. (P–Q) Early L3 eye imaginal disc of control (P) and *ey*>*dMyc* RNAi (Q). Glial cells migrate before the onset of differentiation (shown by Hrp staining) in *ey*>*dMyc* RNAi. (R) Pi3K92E knockdown in the eye disc reduces tissue growth but does not affect glia overmigration. (S) *hth*>*dMyc* RNAi eye discs showing glia overmigration. (T) *repo*>*dcr-2*>*dMyc* RNAi eye discs have reduced numbers of glial cells. Glial cells stained with Repo are shown in red; Hrp or Elav are used to label photoreceptors in grey, and DAPI stains DNA in blue. A dashed line represents the MF. Scale bars correspond to 10 μm.

The eye imaginal disc of *Drosophila* is a good system to study neuron-glia crosstalk because these cell types have distinct and spatially separated origins. Photoreceptors originate from retinal progenitor cells in the eye disc, while glial cells derive from the optic stalk [24]. Glial cells migrate outwards from the brain along the optic stalk, towards the basal surface of the eye disc. This process is tightly coordinated with the ongoing photoreceptor differentiation [24] which takes place behind the morphogenetic furrow (MF), an epithelial indentation that progresses in a posterior to anterior direction (reviewed by [49]). Knocking-down *dMyc* function by dsRNA expression, specifically in eye progenitor cells (but not in glial cells) using the *ey*-Gal4 driver, leads to a reduced eye phenotype (Fig 1A–1C and S1A and S1B Fig), as expected given its role in cell growth regulation [34]. In control eye imaginal discs, perineurial glial cells (PG) migrate from the optic lobes into the eye disc and its migration terminate 3 to 4 ommatidial columns posterior to the MF (Fig 1D and 1F) [50–52]. Unexpectedly in *dMyc* RNAi retinas, glial cells can migrate pass the MF in approximately 90% of the discs (S1E Fig), moving beyond the atonal expression stripe anterior to the MF (Fig 1A–1Q). These cells migrate as multicellular chains or strands and importantly, detachment from other glial cells in the endogenous domain (posterior to the MF) was never observed. Glia overmigration was rescued in 96% of the *ey*>*dMyc* RNAi eye discs by *dMyc* co-expression (S1D and S1E Fig). During normal migration, PG cells stop just posterior to the MF when they contact a nascent photoreceptor axon (R-axon) and initiate differentiation into wrapping glia (Fig 1H) (reviewed by [53]). In *ey*>*dMyc* RNAi retinas, glial cells still differentiate into wrapping glia as detected by the expression of *sprouty* (*sty*) [54] (Fig 1H and 1I) and by the presence of axonal wrapping (Fig 1J, 1K, 1M and 1N). Furthermore, the majority of overmigrating cells do not express *sty* suggesting that these cells are PG and that differentiation into wrapping cells is still dependent on photoreceptor differentiation (only one disc out of the 10 analysed showed *sty* expression in overmigrating glia; Fig 1H and 1I). Glia overmigration is not a response towards patches of ectopic photoreceptor differentiation (Fig 1F and 1G) or axon misrouting in the anterior region, as those are not observed (S1F Fig). To assess whether excessive migration of PG cells is accompanied by changes in glial cell morphology we labelled these cells with LexA-driven expression of CD2-GFP independently of *ey*-Gal4-driven *dMyc* RNAi. In contrast with control glia (Fig 1L), cells on the leading edge of migration in *ey*>*dMyc* RNAi eye discs show very long projections (Fig 1O) that extend anteriorly beyond differentiating photoreceptors (Fig 1J and 1M). dMyc is required for multiple functions in the cell, but a reduction of dMyc levels does not directly trigger significant autonomous apoptotic cell death (S1G Fig, left panel) [55,56]. Preventing residual apoptosis using a H99 deletion mutant which removes the pro-apoptotic genes *reaper*, *hid*, and *grim* [57] was not sufficient to interfere with excessive glia migration in *ey*>*dMyc* RNAi eye discs (S1G Fig, left and middle panel). Furthermore, overexpressing *dMyc* in the eye disc (*dMyc*^OE^) increased apoptotic cell death but did not induced glia overmigration (S1G Fig, right panel). These experiments suggest that in the context of Myc misregulation, apoptosis is not directly regulating the extension of glia migration. Furthermore, *repo*>*dMyc*^*O*E^ caused no overmigration, thus it is not sufficient that glial cells express higher Myc levels in relation to progenitors to migrate excessively (S1H Fig). dMyc-dependent glia overmigration is observed already in the early 3^rd^ instar eye discs (Fig 1P and 1Q), before photoreceptor differentiation initiates. This strongly suggests that the overmigration phenotype results from dMyc depletion in photoreceptor progenitor cells. In fact, knocking-down *dMyc* in differentiated photoreceptors caused no glia overmigration phenotype (S1H Fig), whereas *dMyc* depletion in the anterior region of the disc (using *hth*-Gal4 driver) causes glia to overshoot beyond the MF (Fig 1S). In contrast, specific depletion of *dMyc* in glial cells (using the glia-specific *repo*-Gal4 driver) results in fewer glial cells in the disc (Fig 1T) [58]. In order to understand if the observed glia overmigration phenotype is a consequence of the defective tissue growth induced by Myc depletion, we next knocked down the growth regulator *Pi3K92E* [59]. Decreased levels of Pi3K92E also result in smaller discs, but glia did not overmigrate (Fig 1R). Overall, these experiments suggest that Myc is required in retinal progenitors to control glia migration in a non-autonomous manner.

### dMyc non-autonomous regulation of retinal glia proliferation

**Fig 2:**
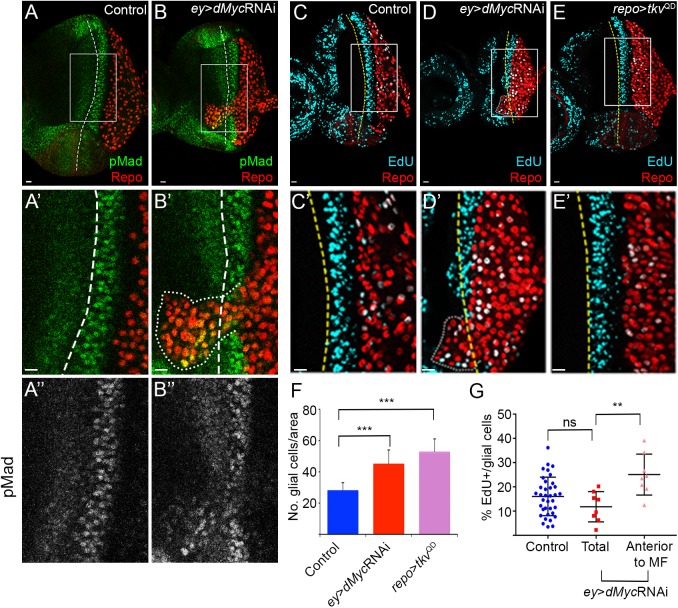
dMyc function in eye progenitors regulates retinal glia pMad activation and proliferation. (A–B) pMad staining (green) in Control (A) and *ey*>*dMyc* RNAi (B). A’ and B’ show magnifications of the inset in A and B. A” and B” show pMad staining in the magnified region. (C–E) EdU staining (light blue) in the control (C), *ey*>*dMyc* RNAi (D) and *repo*>*tkv*^QD^ (constitutively active; E). C’–E’ show magnifications of the inset in C, D and E. The region with higher staining of both pMad and EdU corresponds to the second mitotic wave of photoreceptors differentiation (eye disc cells, not glia). Glial cells stained with Repo are shown in red; A dashed line represents the MF. Scale bars correspond to 10 μm. (F) Graph showing the number of glial cells per area in Control, *ey*>*dMyc* RNAi and *repo*>*tkv*^QD^. (G) Graph showing the percentage of EdU positive glial cells in Control and *ey*>*dMyc* RNAi (total and glial cells anterior to MF).

In *ey*>*dMyc* RNAi we observed an increase in the total number of retinal glial cells (average of 112 in control vs. 153 in *ey*>*dMyc* RNAi; S1C Fig), therefore we next examined the contribution of glial cell proliferation to the overmigration phenotype (Fig 2). The autonomous activation of the Dpp pathway, through activation of the Dpp/TGF-β receptor Thickveins (Tkv), induces an accumulation of glial cells in the eye disc (Fig 2F) [60]. Relatively low levels of Mad activation (phospho-Mad; pMad), the transcription factor acting downstream of Dpp were detected in glial cells of control retinas (Fig 2A and S2 Fig). Interestingly, in *ey*>*dMyc* RNAi eye discs we observed an increased activation of Mad, but mainly in glial cells that migrate beyond the MF and overshoot anteriorly (Fig 2A and 2B). The localised pattern of pMad hyperactivation correlated well with increased EdU staining for cell proliferation in overmigrating cells, but not in the overall glia population where no increased EdU is observed (Fig 2C, 2D and 2G). These experiments indicate that in eye discs with reduced *dMyc* expression, glial cells that pass the MF respond highly to Dpp (expressed normally at the MF) and proliferate. However, on its own, the accumulation of glial cells in the eye disc, when a constitutively active form of Tkv (Tkv^QD^) (Fig 2E) or the constitutively active growth promoter Yorkie [58] are expressed in glia, is not sufficient to promote overmigration beyond the MF. This suggests that other mechanisms are at play in Myc-depleted discs.

### dMyc inhibits JNK activation in the eye imaginal disc

**Fig 3:**
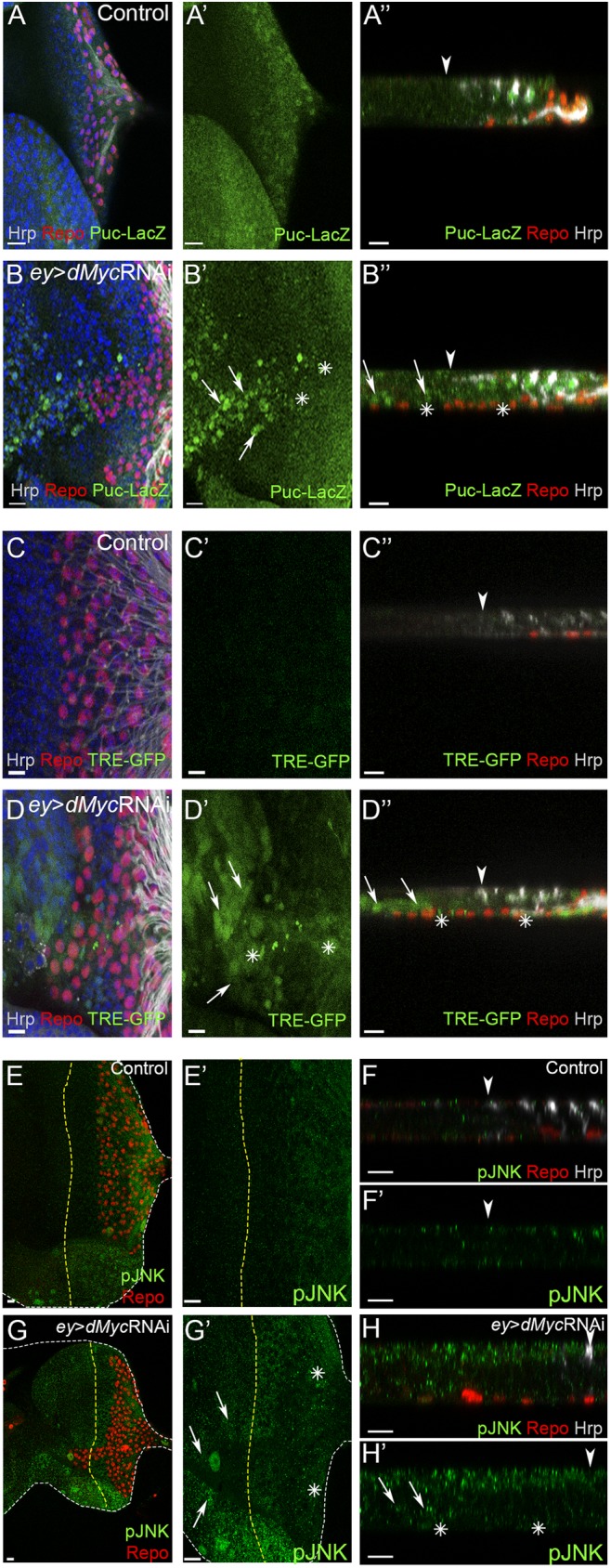
dMyc is required to prevent ectopic JNK pathway activation. (A–B)–*puc*^E69^ expression (β-galactosidase reporter for *puc*; green) in control (A) and *ey*>*dMyc* RNAi (B). (C–D)–TRE-GFP expression (green) in control (C) and *ey*>*dMyc* RNAi (D). (E–H)–pJNK expression (green) in control (E and F) and *ey*>*dMyc* RNAi (G and H). A”,B”, C”,D”, F, F’, H and H’ show transversal views from the eye disc. Arrows point towards eye disc areas with high JNK pathway activation and asterisks represent JNK activation in glia. Glial cells stained with repo are shown in red, Hrp shows the photoreceptors in grey and DAPI stains DNA in blue. A yellow dashed line or arrowhead represents the MF. Scale bars correspond to 10 μm.

**Fig 4:**
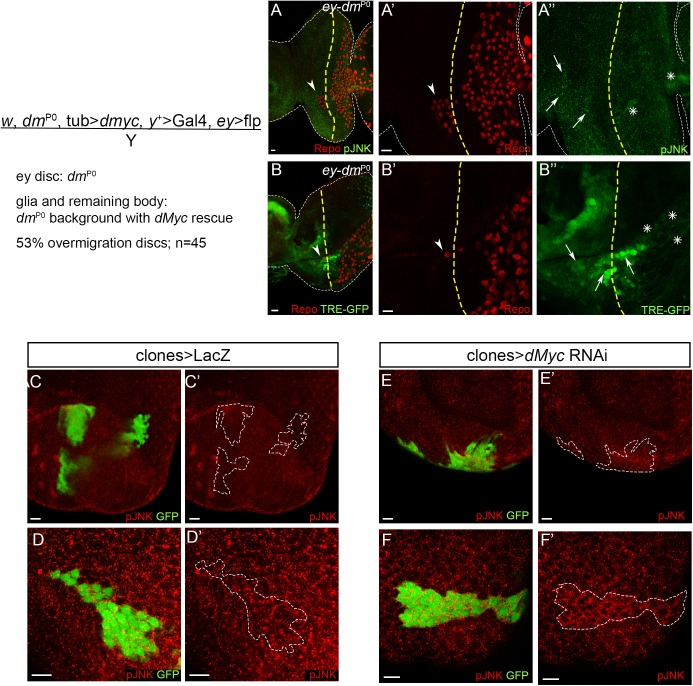
Clonal inhibition of dMyc induces JNK pathway activation. (A and B) Glia overmigration and JNK activation in eye discs mutant for Myc (*ey*-*dm*^P0^) in a phenotypically wild-type animal. (A) pJNK expression (green) in male dMyc mutant eye discs (*ey*-*dm*^P0^). A’ (glia) and A” (pJNK) show a magnification from A. (B) TRE-GFP expression (green) in male dMyc mutant eye discs (*ey*-*dm*^P0^). B’ (glia) and B” (TRE-GFP) show a magnification from B. Arrowheads point towards glia overmigration (beyond the MF). Arrows indicate eye disc areas with high JNK pathway activation and asterisk represent JNK activation in glia. Glial cells stained with repo are shown in red. A yellow dashed line represents the MF. Scale bars correspond to 10 μm. (C–F) Control (C and D; LacZ) or dMycRNAi (E and F) clones were induced in the Drosophila early eye disc 48 ± 24 hours after egg laying (AEL). Images show representative clones in the epithelial layers of the disc proper (C and E) and peripodial epithelium (D and F), marked positively by the presence of GFP. pJNK is shown in red. A dashed white line surrounds the clone positive region. Scale bars correspond to 10 μm.

The JNK signalling pathway has been widely implicated in morphogenetic and cell migration regulation [61,62]. Recently, JNK activity was shown to be important for neuron-glia crosstalk upon neuronal damage in the *Drosophila* embryonic CNS [63], *Drosophila* developmental axon pruning [64] but also in mammalian Schwann cells in response to axonal injury [65,66]. Thus, we tested if Myc function in limiting the extent of glia migration is associated to a role in modulating JNK activation during retinal development. We analysed JNK activation using two transcriptional reporters: an enhancer trap line, *puc*^E69^-LacZ for the JNK target Puckered [67], and TRE-GFP that is under the control of AP-1 binding sites for JNK transcriptional effectors Jun/Fos [68]. There was a strong increase of *puc*-LacZ (Fig 3A and 3B) and TRE-GFP expression (Fig 3C and 3D) in the eye disc proper upon *dMyc* knockdown, especially at the anterior region of the disc (Fig 3A–3D arrows). Significantly, we observed a similar upregulation of JNK pathway activation in the anterior domain using an antibody against phosphorylated JNK (activated JNK–pJNK; Fig 3E–3H). We also detected weak ectopic activation of the JNK pathway in glia (using *puc*^E69^-LacZ, TRE-GFP and pJNK; Fig 3A–3H asterisks), suggesting that Myc-depleted eye imaginal discs can induce non-autonomous glia JNK activation. Importantly, we also observed glia overmigration and pJNK activation in eye discs mutant for Myc (*ey*-*dm*^*P0*^) generated by *ey*-*flp* mediated removal of a rescuing *dMyc* transgene. In this experiment, glial cells are phenotypically wild-type (Fig 4A and 4B) [69]. Furthermore, in third-instar eye imaginal discs we observed pJNK autonomous upregulation in some of the *dMyc* RNAi mitotic *flp*-out clones, when induced at 48-72h AEL (Fig 4C–4F). The JNK upregulation in *dMyc* loss-of-function was surprising as it has previously been reported that JNK can regulate MYC through phosphorylation [70], ubiquitination and degradation [71], but never the opposite.

### JNK is required downstream of dMyc to induce glia overmigration

**Fig 5:**
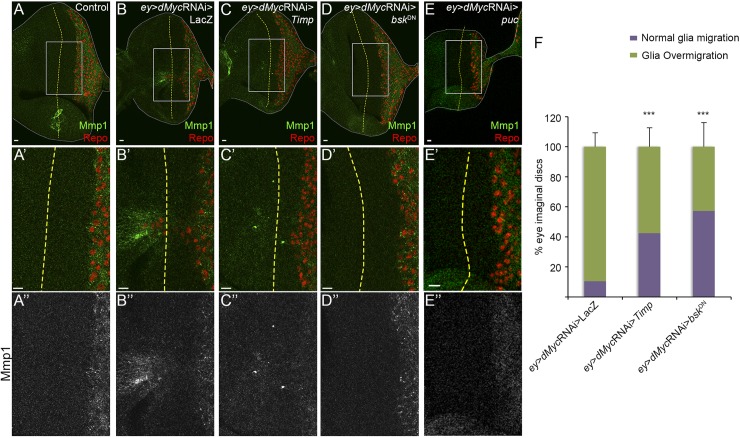
Glia overmigration induced by dMyc knockdown in the eye disc is triggered by JNK and mediated by Mmp1 expression. (A–E) Mmp1 expression (green) in control (A), *ey*>*dMyc* RNAi>LacZ (B), *ey*>*dMyc* RNAi >*Timp* (C), *ey*>*dMyc* RNAi>*bsk*^DN^ (D) and *ey*>*dMyc* RNAi>*puc* (E). A’–E’ shows magnification of the inset shown in A–E respectively. A”–E” show Mmp1 expression. (F) Graph showing the percentage of eye imaginal discs with glia overmigration vs. normal glia migration in *ey*>*dMyc* RNAi>LacZ (n = 148), *ey*>*dMyc* RNAi>*Timp* (n = 146) and *ey*>*dMyc* RNAi>*bsk*^DN^ (n = 110).

Next, we analysed if the activation of the JNK pathway was important for glia overmigration. When *dMyc* expression is knocked down in the eye disc there is a significant Mmp1 upregulation, hallmark of JNK activation [72], close to the overshooting glial cells (Fig 5A and 5B; S3A and S3B Fig). MMPs are key players in tissue remodelling through their ability to cleave ECM constituents and to regulate the function of transmembrane proteins. To inhibit MMPs proteolytic activity we overexpressed *Timp* (tissue inhibitor of metalloproteinase) that is able to interact with MMPs Zn-binding motif [73]. Co-expression of *Timp* with *dMyc* RNAi, using *ey*-Gal4, inhibited glia overmigration without interfering with normal glia migration in the posterior domain of differentiating photoreceptors (*Timp* co-expression rescues overmigration in 62 out of 146 eye discs analysed; in *dMyc* RNAi only 14 out of 148 discs stop glia migration before the MF; Fig 5A–5C and 5F). *dMyc* mutant eye discs (*ey*-*dm*^P0^/Y) in a heterozygotic *Mmp1* mutant genetic background also show a rescue of overmigration (S3C and S3D Fig). In addition, inhibition of the JNK pathway in *dMyc* RNAi eye discs by overexpressing a dominant-negative form of Basket (*Bsk*^DN^, *Drosophila* JNK) [74] or the Puckered protein (a phosphatase that inhibits Bsk [75]; Fig 5D and 5E respectively) rescued glia overmigration in the majority of the eye discs analysed (overexpression of *bsk*^DN^ rescues overmigration in 62 out of 146 eye discs analysed; Fig 5F). As expected, downregulation of the JNK pathway in *ey*>*dMyc* RNAi background prevented Mmp1 upregulation (Fig 5A”–5E”). Interestingly JNK pathway inhibition in *ey*>*dMyc* RNAi by *bsk*^DN^ does not change the proliferation of glial cells (S4 Fig). These experiments suggest that inhibition of dMyc in the proliferative non-differentiated region of the eye disc causes JNK pathway activation, primarily in the eye disc proper, and Mmp1 upregulation. This has a non-autonomous effect in glia allowing its migration to the retinal progenitors region (overcoming its normal boundary).

### dMyc knockdown induces ECM remodelling

**Fig 6:**
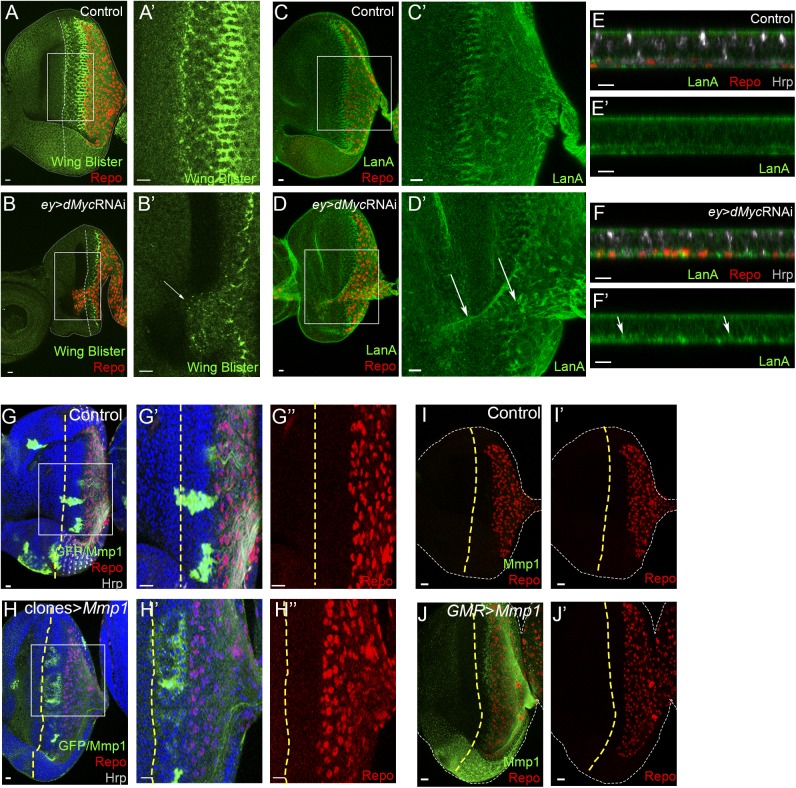
ECM remodelling by Mmp1 expression in the eye disc is not sufficient to trigger glia overmigration. (A and B) Wing Blister expression (green) in control (A) and *ey*>*dMyc* RNAi (B). A’ and B’ show a magnification from Wing Blister expression of the inset on A and B respectively. Arrow points towards ectopic Wing Blister expression. (C–F) Laminin A (LanA) expression (green) in control (C and E) and *ey*>*dMyc* RNAi (D and F). C’ and D’ show a magnification from LanA expression of the inset on C and D respectively. E and F show transversal sections from the eye disc where a punctated enrichment of LanA deposition is detected. Arrow points towards enrichment of LanA deposition. (G and H) Clonal overexpression of LacZ (control; G) and *Mmp1* (H). G′, G″, H′ and H″ show magnifications of the inset shown in G and H respectively. Clones are marked positively by the presence of GFP (concomitant Mmp1 staining in green). Hrp stains the photoreceptors in grey. (I and J) Control (I) and *Mmp1* overexpression (J) in differentiated photoreceptors (with *GMR*-Gal4). Mmp1 (green) normal low levels at the posterior region are not visible due to a change in the settings to accommodate the high Mmp1 expression in J. Repo (glia) is shown in red. A yellow dashed line represents the MF. Scale bars correspond to 10 μm.

Glial cell migration in the CNS and PNS have been shown to require significant extracellular matrix (ECM) modulation [76–79]. In particular, we have previously shown that integrins are important for glial cell migration and Laminin A rearrangement [4]. Additionally it was previously reported that Laminin W is required for cell adhesion and migration during embryonic and imaginal development [80,81]. As we observed Mmp1 upregulation in *ey*>*dMyc* RNAi (Fig 5A and 5B), we decided to assess if changes in ECM components or regulators are associated to glia migration in these eye discs. In *Drosophila*, two laminin trimers have been described: laminin A (α3,5; β1; γ1) and laminin W (α1,2; β1; γ1). *Wing blister* (*wb*) encodes a α-Laminin 1,2 protein (subunit of Laminin W) that in control eye discs accumulates in a column posterior to the MF, near the limit for glial cell migration. When *dMyc* RNAi was expressed in the eye disc, glial cells that overshoot the MF co-localise with regions of ectopic high WB levels (Fig 6A and 6B). LamininA (LanA; *Drosophila* α-Laminin 3,5 protein) subunit deposition is even more widespread accumulating in *puncta* both in the glia posterior domain, and in the overmigrating glia (Fig 6C–6F). Next, we investigated if tissue ECM remodelling mediated by Mmp1 upregulation was sufficient to explain glial cells overmigration in *ey*>*dMyc* RNAi. Clonal overexpression of Mmp1 in the eye disc (Fig 6G and 6H) or in differentiated photoreceptors using *GMR*-Gal4 (Fig 6I and 6J) was not sufficient to allow glia to overmigrate. Furthermore, inhibiting MMPs expression or activity in glial cells (by overexpressing Timp, and Mmp1 and Mmp2 RNAi) did not interfere with the normal glia migration pattern (S3E–S3I Fig). Thus, Mmp activity appears to have a limited role in allowing excessive glia migration in the eye disc, as it is necessary but not sufficient for this phenotype.

### JNK activation in the eye disc is sufficient to induce glia overmigration

**Fig 7:**
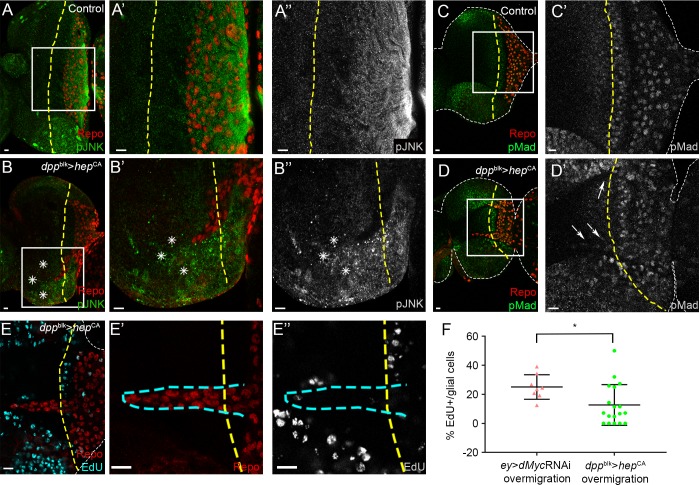
JNK activation in the eye disc is sufficient to induce non-autonomous glia overmigration. (A and B) pJNK expression (green) in Control (A) and *dpp*^blk^>*hep*^CA^ (B). Asterisk show increased pJNK expression. (C and D) pMad staining (green) in Control (C) and *dpp*^blk^>*hep*^CA^ (D). Arrows point towards overmigrating glia that does not show pMad activation. A’–D’ and A”–B” show a magnification from the inset on figure A to D respectively. (E) EdU staining (light blue) in *dpp*^blk^>*hep*^CA^. E’ show magnification of overmigrating glia in red and E” show a magnification of EdU staining in grey. Blue dashed line surrounds overmigrating glia. Glial cells stained with repo are shown in red. A yellow dashed line represents the MF. Scale bars correspond to 10 μm. (F) Graph showing the percentage of EdU positive glial cells in Control in *dpp*^blk^>*hep*^CA^ total and *dpp*^blk^>*hep*^CA^ overmigration (glia anterior to MF).

**Fig 8:**
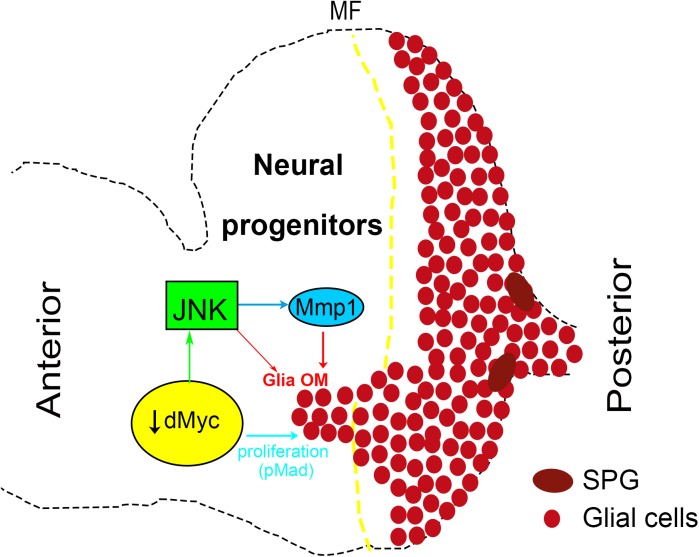
A model for glia overmigration when retinal progenitors fitness is compromised by loss of dMyc. Decreased levels of dMyc in retinal progenitors induce JNK activation and Mmp1 expression in eye disc cells. In a non-autonomous manner glia overmigrates anteriorly to the MF where it displays increased proliferation in response to autonomous pMad activation. JNK activation in the eye disc *per se* is sufficient to produce Mmp1 and induce glia overmigration but not to stimulate glia proliferation.

Having shown an essential role for JNK activity in glia overmigration induced by *dMyc* RNAi, the next question we addressed was if JNK activity *per se* is sufficient to induce this phenotype. We overexpressed constitutively active *hemipterous* (*hep*^CA^) in the eye disc driven by *dpp*blk-Gal4, to avoid *ey*-Gal4-driven lethality (S5A and S5B Fig). Hep is homologous to Jun kinase kinase (JNKK) [82], and activates Bsk (*Drosophila* JNK) through phosphorylation. As expected, we observed increased upregulation of the JNK activation by phosphorylation (pJNK; Fig 7A and 7B). Interestingly, JNK activation in the eye disc was sufficient to induce glia overmigration (Fig 7A–7E). The same was verified using the *Optix*-Gal4 driver (S5D Fig) and in the few larvae that survived from *ey*-Gal4 and A4-Gal4 driven *hep*^CA^ (S5A–S5C Fig). *ey*>*dMyc* RNAi overmigration is accompanied of pMad activation and increased proliferation in overmigrating glia (Fig 2) so we analysed if this activation was mediated by JNK activation. Surprisingly, no Dpp/TGF-β signalling activation (Fig 7C and 7D) or increased proliferation (Fig 7E and 7F) was detected, for what we conclude that ectopic glia localization anterior to the MF is not sufficient to activate Dpp/TGF-β signalling and promote proliferation. pMad activation occurs downstream of Myc-knockdown but JNK activation is not sufficient to promote it (Fig 8). Furthermore, JNK-mediated glia recruitment requires a non-autonomous mechanism, since modulation of the JNK pathway in glia does not appear to have a major role in glia migration (S5E to S5J Fig). Hep activation in the differentiated photoreceptors also does not affect the glia migration pattern (S5K Fig). We can conclude that JNK activation in eye progenitors is sufficient to non-autonomously induce overmigration of glial cells into the eye disc.

## Discussion

### dMyc non-autonomous effect in glia

Growth requires energy as well as protein synthesis and MYC activity plays an important role in controlling metabolic pathways such as glycolysis and glutaminolysis [43,83,84]. Hypomorphic dMyc mutant cells are characterized by a reduced growth rate and correspondingly smaller size, with proliferation rates being affected when dMyc levels are strongly reduced [38]. In here, we investigated if decreased cellular fitness in eye progenitors caused by reducing *Drosophila* Myc expression trigger non cell-autonomous responses in retinal glia development. Indeed, glial cells sense the decreased dMyc levels in retinal progenitors and respond with increased migration and proliferation (Fig 8). This phenomenon is non-autonomous, as when dMyc is depleted in glial cells it does not induce glia overmigration. Cell competition [55,85,86] was observed in mutant dMyc clones that were still functional but were culled by programmed cell death [55] if ‘‘fitter” cells were present that could replace them. We found no evidence for neurons and glia to be comparing their Myc levels in the context of overmigration, as reducing dMyc expression in differentiated photoreceptors or overexpressing dMyc in glia did not change the normal glia migration pattern.

The Dpp/TGF-β pathway stimulates glia proliferation [58,60] and in fact we observed activation of Mad and increased proliferation in overmigrating glia (Fig 2). However, activating Dpp signalling in an autonomous fashion in all retinal glial cells (*repo*>*tkv*^QD^) was not sufficient to stimulate migration of glia. Overmigration of retinal glial cells has been observed in a few genetic backgrounds, however the patterns of glia migration and proliferation in Myc-depleted eye discs are quite distinct. For example, when the Hh effector Ci is expressed in glial cells they overmigrate being followed by axonal misrouting [60], which was not observed in dMyc-depleted retinas (Fig 1J and 1M and S1F Fig). In dMyc-depleted eye discs, glial cells do not migrate towards patches of ectopic neurons nor are accompanied by axon misrouting as has been described for other contexts of alterations in glia migration [60,87]. If Spinster, a transmembrane protein localizing to the late endosome or lysosome, is downregulated in glia there is overmigration [88], but this is accompanied by increased glia proliferation that is restricted to the optic stalk, unlike the increased proliferation of overmigrating glia that overshot the MF in dMyc-knockdown eye discs. Pvr (PDGF receptor homologue)-activated glia also overmigrate, but distinctly from *ey*>*dMyc* RNAi, through both the basal and apical sides of the eye disc and without affecting glia proliferation [78]. Additionally, retinal glial cells overmigrating in response to dMyc loss-of-function in eye disc progenitors are still able to activate Sty-LacZ (a marker of wrapping glia) unlike overmigrating glial cells overexpressing Cut, a transcription factor activated by Dref and FGF [89].

Interestingly, we have shown that *dMyc*-RNAi eye discs show autonomous activation of JNK and ectopic Mmp1 expression, possibly associated to ECM remodelling. In hemocytes, JNK has been implicated in cell migration through Mmp secretion and Pi3K92E activation [90]. A different mechanism is in place in dMyc-dependent retinal glia overmigration, as *dMyc* RNAi does not induce ERK activation in migrating glia (S6A and S6B Fig) nor Pi3K92E activation in glia is sufficient to induce dMyc-like glia migration (S6C and S6D Fig). Autonomous JNK activation has been shown to lead to a propagation loop, mediated by upregulation of the *Drosophila* Eiger/TNF (Egr) that activates JNK in neighbouring cells [91]. We excluded a similar retinal progenitors-glia communication through JNK-mediated Egr expression, as manipulation of Egr levels did not change the glia overmigration phenotype (S6E–S6G Fig). Importantly, JNK activation is both necessary and sufficient to induce non-autonomous glia overmigration. However, we did not observe increased glia Mad activation when glia overmigration was induced by JNK activation in the eye disc and in this genotype we also did not observe increased proliferation of glial cells that overshoot the MF. Thus, we suggest that in *ey*>*dMyc* RNAi eye discs, glial cells that reach the anterior domain are highly responsive to Dpp signal but this response is not intrinsic to glia overmigration, requiring dMyc loss-of-function.

Our results show that dMyc has a role in limiting JNK activation in undifferentiated eye disc cells. Thus, it will be interesting to address if dMyc has a general role in JNK repression through direct transcriptional regulation of JNK pathway members. Available ChIP-sequencing data shows that *puc* and *Dusp10* (the mammalian orthologue of *puc*) are directly bound by MYC [92–95], suggesting that MYC could regulate JNK signalling via direct control of *puc*/*Dusp10* transcription.

In the absence of dMyc in the eye, glial cells overmigrate as a chain (distinctly from the single cell migration mode [96]) suggesting that the leading tip cell could be competing for external signals such as FGF [54,97]. This could be the case as FGF is important for normal glia migration and differentiation [54,98]. Importantly, we confirm that depletion of *bnl* function in the eye disc causes non-autonomous stimulation of glia migration [98] and that this is accompanied by activation of Mmp1 and pMad in the anterior domain, and glial cells proliferation, in a similar manner to dMyc knockdown (S8 Fig). However, we failed to observe a strong genetic interaction between *bnl* and *dMyc*, suggesting that distinct changes in the context of growth potential of retinal progenitors could converge on JNK activation.

### JNK activation in eye disc progenitors regulates glia overmigration

dMyc-depleted eye discs induce glia proliferation and overmigration, characteristics of many types of tumors, including glioblastomas. In recent years it has become more evident that tumour formation involves interactions between the tumour-initiating cells and the tumour microenvironment niche, all of which contribute to the tumour proliferative and invasive capacity [99]. In this work we show that the dMyc-depleted microenvironment (retinal progenitor cells) activate a stress pathway triggering epithelial/ECM changes that are actively interpreted by glia. This stress response is triggered by JNK activation and mediated by Mmp1 expression. JNK is indispensable for both cell proliferation and apoptosis depending on the cell type, the nature of the upstream stimulus, the duration of its activation and the activities of other signalling pathways [100,101]. We show that JNK activation induced by *dMyc* loss-of-function in *Drosophila* eye was not caused by or result in apoptosis, demonstrating a non-apoptotic role for *Drosophila* JNK in this context. Activation of JNK in eye progenitors was sufficient to activate Jra/Kayak (c-Jun/c-fos homologues) and trigger non-autonomous glia migration. Interestingly, JNK function in developmental axon pruning and injured axons has been described either as deleterious, through the induction of axonal degradation [102–106] or beneficial, through the activation of axonal regeneration [63,64,107–109]. JNK activation is essential for phagocytosis and autophagy both in *Drosophila* glia [63,110], mammalian astrocytes [111], Schwann cells [66,112,113], microglia [114] as well as in non-glial cells types [115,116]. We envisage that overmigrating glia in *Drosophila* eye discs mimics reactive gliosis in mammals (important mechanism of rescue/confinement of a brain injured area) [117,118]. Thus overmigrating glia might play roles in promoting tissue regeneration through its phagocytic activity and possible secretion of growth and/or proliferation factors. Undoubtedly, future studies are required to characterise the roles of retinal dMyc in modulating glia responses. Overall, we describe here a useful system to further understand the mechanisms and functional roles of glia activation in response to perturbations of photoreceptor neuronal development.

## Materials and methods

### Fly husbandry

Most crosses were raised at 25°C under standard conditions. The following stocks (described in FlyBase, unless stated otherwise) were used: *ey*-Gal4, *repo*-Gal4, *dppblk*-Gal4, *hth*-Gal4 [119], *GMR*-Gal4, *elav*-Gal4, *optix*-Gal4, A4-Gal4, 3’*atonal*1.2-pGWGFP [120], UAS-lacZ, *sprouty*-LacZ [54], *repo*LexA-LexAopCD2-GFP [54], pucE69 [75], (#109029, Kyoto), TRE-GFP [68], Df(3L)H99 (#1576), w1118, UAS-*dMyc*, UAS-*Pi3K92E*^CAAX^, UAS-CD8GFP. UAS-*egr* strong allele [121], UAS-dicer-2, UAS-CD4tdTOM, UAS-*Pi3K92E* RNAi (#27690 and #35798), UAS- *tkv*^QD^ (#36536), UAS-*Timp* (#58708), UAS-*bsk*^DN^k53R (#9311), UAS-*puc* [75], UAS-*hep*^CA^ (#9305), UAS-*Mmp1* RNAi (VDRC #101505), UAS-*Mmp2* RNAi (VDRC #107888), *Mmp1*^2^, *Mmp1*^K04809^, UAS- *egr* RNAi (#55276), UAS- *bnl* RNAi (VDRC #5730), *ey*-*dm*WT = *yw dm*+ *tubulin*-FRT-*dMyc*-cDNA-FRT-Gal4, *ey*-*flp*/Y, *ey*-*dm*^P0^ = *yw dm*^P0^ *tubulin*-FRT-*dMyc*-cDNA-FRT-Gal4, *ey*-*flp*/Y [69], UAS-*dMyc* RNAi (VDRC #2948). The RNAi was validated by testing other lines: Bloomington #5784, VDRC #v2947 and VDRC #106066.

Mitotic recombination was induced using the FLP/FRT method. dMyc knockdown clones, or control clones overexpressing LacZ, were induced by heat shock (45 min at 37°C) at 48 ± 24 hours after egg laying (AEL) in larvae of the genotype *yw* hs*flp*/+; *act*>*y*+>Gal4, UAS-GFP/UAS-*dMyc*RNAi/+ and *yw* hs*flp*/+; *act*>*y*+>Gal4, UAS-GFP/+; UAS-LacZ/+.

### Immunohistochemistry

Eye-antennal imaginal discs were dissected in cold Phosphate Buffer Saline (PBS) and fixed in 3.7% formaldehyde/PBS for 20 minutes. Immunostaining was performed using standard protocols. Primary antibodies used were: mouse anti-Repo antibody at 1:10 (8D12, Developmental Studies Hybridoma Bank, DSHB), rat anti-repo (1:5000, gift from Dr. Benjamin Altenhein, Institut für Genetik, Germany), rabbit anti-repo (1:25000, gift from Dr. Benjamin Altenhein), rat anti-Elav 1:100 (7E8A10 DSHB), mouse anti-Elav antibody at 1:50 (9F8A9, DSHB), rabbit anti-pMad (P-Smad1/5, Ser463/465) antibody at 1:100 (9516, Cell Signaling), rabbit anti-pJNK antibody at 1:200 (V793A, Promega), mouse anti-Mmp1 antibody at 1:25 (1:1:1 of 14A3D2, 3A6B4 and 5H7B11 all from DSHB), rabbit anti-Wb antibody at 1:30 (kind gift from Stefan Baumgartner, Lund University, Sweden), rabbit anti-pERK antibody (Phospho-p44/42 MAPK) at 1:200 (4370, Cell Signaling), rabbit anti-pH3 antibody at 1:1000 (Upstate), goat anti-HRP antibody Cy5-conjugated at 1:100 (323-175-021, Jackson ImmunoResearch), rabbit anti-dMyc at 1:100 (sc-28207, Santa Cruz) rabbit anti-cleaved Caspase-3 at 1:200 (9661, Cell Signaling), rabbit anti-β-galactosidase antibody 1:2000 (Cappel, 55976, MP Biomedicals). Appropriate Alexa Fluor conjugated secondary antibodies used were from Molecular Probes. For Ethynyl deoxyuridine (EdU) experiments, dissected eye-antennal imaginal discs were incubated in 20 μM EdU/PBS for 20 minutes, at room temperature, washed with PBS and fixed as described above. Alexa Fluor Azide detection was performed according to Click-iT EdU Fluor Imaging Kit (Invitrogen).

Images were obtained with the Leica SP5 confocal system and processed with Adobe Photoshop.

Glial cells were counted and eye discs measured in Fiji. Mean and standard deviation were calculated for each case. Results from glial cell counting were analysed by unpaired Student’s t test. Results from overmigration discs were analysed by Fisher’s exact test in contingency tables. p-values are shown with numbers or with the following asterisk code: * = p<0.05; ** = p<0.01;*** = p<0.001;**** = p<0.0001. Error bars present in all graphs represent the standard deviation.

## Supporting information

S1 Fig Glia overmigration caused by dMyc depletion in eye progenitors is independent of apoptosis. (A) dMyc expression in Control and *ey*>*dMyc* RNAi.
(B) Graph showing the eye disc area (arbitrary units) in control (n = 37) and *ey*>*dMyc* RNAi (n = 40).
(C) Graph showing the total number of glial cells in control (n = 13) and *ey*>*dMyc* RNAi (n = 13) eye discs (10 to 15 rows of photoreceptor differentiation).
(D) dMyc overexpression (*ey*>*dMyc* RNAi>*dMyc*) rescues glial overmigration in *ey*>*dMyc* RNAi. Glia is shown in red.
(E) Graph showing the percentage of eye imaginal discs with glia overmigration vs normal glia migration in *ey*>*dMyc* RNAi>LacZ (n = 148) and *ey*>*dMyc*RNAi>*dMyc* (n = 69).
(F) Hrp staining (grey) showing proper axon pathfinding towards the optic stalk in control and *ey*>*dMyc* RNAi. Right panel show a magnification of the inset from middle panel.
(G) Cleaved caspase-3 (Casp) staining (green) in *ey*>*dMyc* RNAi (left panel), *ey*>*dMyc* RNAi; Def (H99)/+ (middle panel) and *ey*>*dMyc*^OE^ (right panel).
(H) Effects of cell-specific dMyc misregulation in glial overmigration: increasing (*dMyc*^OE^) and decreasing (*dMyc* RNAi) levels of dMyc were analyzed in glia (with *repo*-Gal4), the eye disc progenitors (*ey*-Gal4) and differentiated photoreceptors (*elav*-Gal4 and *GMR*-Gal4).
Glial cells are stained with Repo (red) and DNA is counterstained by DAPI (blue). A yellow dashed line represents the MF. Scale bars correspond to 10 μm.
(TIF)

S2 Fig TGF-β activation in retinal glia. Control eye imaginal discs showing TGF-β activation (pMad). Left panel show glial cells stained with Repo in red. Middle and right panels shows pMad in green. Right panel show a magnification from the inset in the middle panel. A yellow dashed line represents MF. Scale bars correspond to 10 μm.
(TIF)

S3 Fig ECM alterations are not sufficient to induce glia overmigration. (A and B) Mmp1 expression (green) in control (A) and *hth*>*dMyc* RNAi (B).
(C) Glia migration in Control (*ey*-*dm*WT), *dMyc* mutant male eye disc (*ey*-*dm*^P0^) and *dMyc* mutant male eye disc heterozygous for Mmp1 mutant–Mmp1^2^ (*ey*-*dm*^P0^;*Mmp1*^2^/+) and *Mmp1*^K04809^ (*ey*-*dm*^P0^;*Mmp1*^K04809^/+). The larvae body (including glia) are *dm*^P0^ mutant rescued with dMyc.
(D) Graph showing the percentage of eye imaginal discs with glia overmigration vs normal glia migration of the genotypes described on C.
(E and F) Mmp1 expression (green) in the Control (E) and *repo*>UAS-*Timp* (F). E’ and F’ show Repo magnifications of the inset in E and F while E” and F” show Mmp1 magnification of the same insets.
(G–I) When compared with the control (G), downregulation of *Mmp1* (H) and *Mmp2* (I) in glia (with *repo*-Gal4) does not interfere with glia migration.
Glial cells stained with Repo are shown in red and DAPI stains the nuclei in blue. A yellow dashed line represents the MF. Scale bars correspond to 10 μm.
(TIF)

S4 Fig Inhibition of JNK prevents glia overmigration response to dMyc depletion. (A–C). EdU staining (light blue) of the control (A), *ey*>*dMyc* RNAi>LacZ (B) and *ey*>*dMyc* RNAi>*bsk*^DN^ (C). A’–C’ show magnifications of the square inset in A–C. A”–C” show EdU staining magnifications in light blue of the same insets. The region with higher staining of EdU corresponds to the second mitotic wave of photoreceptors differentiation.
Glial cells stained with Repo are shown in red; A yellow dashed line represents the MF. Scale bars correspond to 10 μm.
(D) Graph showing the percentage of EdU positive glial cells in Control, *ey*>*dMyc* RNAi>LacZ and *ey*>*dMyc* RNAi>*bsk*^DN^.
(TIF)

S5 Fig Autonomous and non-autonomous roles of JNK in glia. (A–D) Early L3 Control (A) and activation of *hep* (*hep*^CA^) with *ey*-Gal4 (B), A4-Gal4 (C) and *optix*-Gal4 (D).
(E and F) Control (E) and UAS-*hep* (F) overexpression in glia (*repo*4.3-CD8GFP-Gal4). Glial cell membranes are visible in green through the expression of CD8-GFP.
(G and H) Control (G) and *bsk* RNAi in glia (*Dcr2;repo*-Gal4; H) do not affect glia migration.
(I and J) Control (I) and overexpression of *bsk* dominant negative (*bsk*^DN^; J) in glia (*repo*-Gal4) show the same migration pattern of glia as the Control.
(K) Photoreceptors and glia view of Control and *GMR*>*hep*^CA^ showing normal glia migration.
Glial cells stained with Repo are shown in red and DAPI stains the nuclei in blue. Photoreceptors (Hrp) are shown in grey. A yellow dashed line represents the MF. Scale bars correspond to 10 μm.
(TIF)

S6 Fig Evaluation of the contributions of ERK, Pi3K92E, and Eiger to dMyc-associated glia overmigration. (A and B) pERK staining (green) in Control (A) and *ey*>*dMyc* RNAi (B). A’ and B’ show pERK staining and A” and B” show a magnification of the inset represented in A and B respectively.
(C and D) Control (C) and Pi3K92E activation in glia using *repo*4.3-CD8GFP>*Pi3K92E*^CAAX^ (D). Glial cell membranes are detected in green by CD8GFP expression.
(E–G) analysis of Egr role in glia overmigration in *ey*>*egr* (E); *ey*>*dMyc* RNAi>*egr* RNAi (F) and *ey*>*dMyc* RNAi>egr (G). E’, F’ and G’ show Repo staining.
Glial cells are stained with Repo (red), photoreceptors with Hrp (grey) and DAPI counterstains DNA showing the nuclei. A yellow dashed line represents the MF. Scale bars correspond to 10 μm.
(TIF)

S7 Fig FGF/bnl depletion in the eye disc induces JNK mediated glia overmigration. (A–D) Control (A); *bnl* RNAi in the eye disc with *ey*-Gal4 (B), in glia with *repo*-Gal4 (C) and in the anterior domain of the disc with *hth*-Gal4 (D).
(E and F) Transversal analysis of Control (E) and *ey*>*bnl* RNAi (F). Photoreceptors are shown by Elav staining in light blue.
(G and H) Proliferation analysis of Control (G) and *ey*>*bnl* RNAi (H) by pH3 (green). G’, G”, H’ and H” show magnifications of the insets in G and H.
(I and J) pMad staining (green) of Control (I), *ey*>*bnl* RNAi (J). I’, I”, J’ and J” show magnifications of I and J respectively.
(K–M) Mmp1 staining (green) of Control (K), *ey*>*bnl* RNAi>LacZ (L) and *ey*>*bnl* RNAi >*bsk*^DN^ (M). K’, K”, L’, L”, M’ and M” show magnifications of K, L and M respectively.
(N and O) Puc-LacZ expression analysis in *puc*^E69^ Control (N) and *puc*^E69^; *ey*>*bnl* RNAi (O).
(P) Percentage of eye discs with glia overmigration in Control, *ey*>*bnl* RNAi and *hth*>*bnl* RNAi.
(Q) Proliferation rate of glia (by pH3) in Control, *ey*>*bnl* RNAi and *ey*>*bnl* RNAi overmigrating glia (anterior to the MF).
(R) Percentage of eye discs with glia overmigration and normal glia migration in *ey*>*bnl* RNAi>LacZ and *ey*>*bnl* RNAi>*bsk*^DN^.
Glial cells stained with Repo are shown in red and DAPI stains the nuclei in blue. A yellow dashed line represents the MF. Scale bars correspond to 10 μm.
(TIF)

S8 Fig (TIF)
