# Supplementary figures and images for: dMyc is required in retinal progenitors to prevent JNK-mediated retinal glial activation
Source: PLoS Genet. 2017 Mar 7;13(3):e1006647. doi: 10.1371/journal.pgen.1006647 (PMC5360344; doi:10.1371/journal.pgen.1006647)

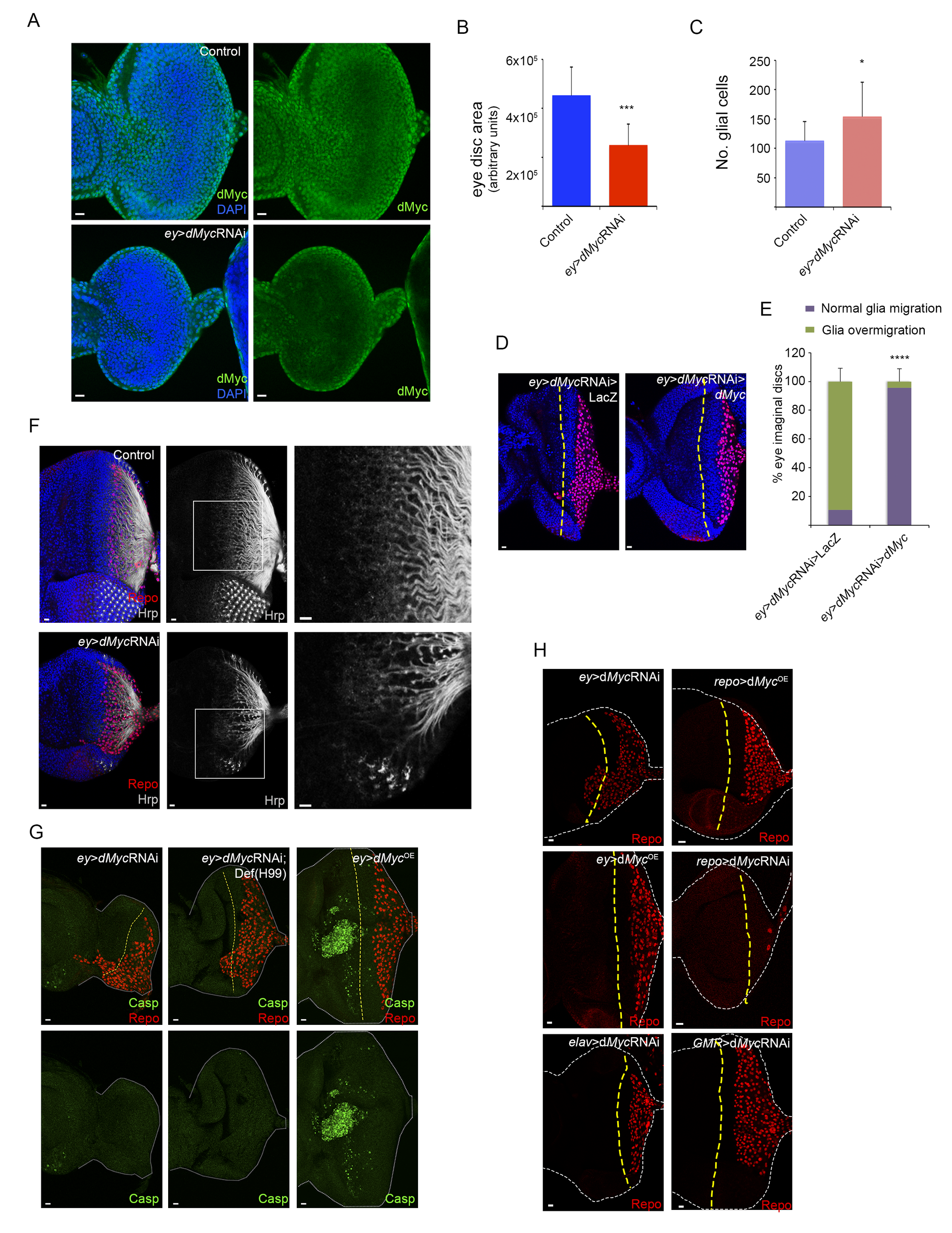

Supplement: S1 Fig — (A) dMyc expression in Control and ey>dMyc RNAi. (B) Graph showing the eye disc area (arbitrary units) in control (n = 37) and ey>dMyc RNAi (n = 40). (C) Graph showing the total number of glial cells in control (n = 13) and ey>dMyc RNAi (n = 13) eye discs (10 to 15 rows of photoreceptor differentiation). (D) dMyc overexpression (ey>dMyc RNAi>dMyc) rescues glial overmigration in ey>dMyc RNAi. Glia is shown in red. (E) Graph showing the percentage of eye imaginal discs with glia overmigration vs normal glia migration in ey>dMyc RNAi>LacZ (n = 148) and ey>dMycRNAi>dMyc (n = 69). (F) Hrp staining (grey) showing proper axon pathfinding towards the optic stalk in control and ey>dMyc RNAi. Right panel show a magnification of the inset from middle panel. (G) Cleaved caspase-3 (Casp) staining (green) in ey>dMyc RNAi (left panel), ey>dMyc RNAi; Def (H99)/+ (middle panel) and ey>dMycOE (right panel). (H) Effects of cell-specific dMyc misregulation in glial overmigration: increasing (dMycOE) and decreasing (dMyc RNAi) levels of dMyc were analyzed in glia (with repo-Gal4), the eye disc progenitors (ey-Gal4) and differentiated photoreceptors (elav-Gal4 and GMR-Gal4). Glial cells are stained with Repo (red) and DNA is counterstained by DAPI (blue). A yellow dashed line represents the MF. Scale bars correspond to 10 μm. (TIF) [file pgen.1006647.s001.tif]

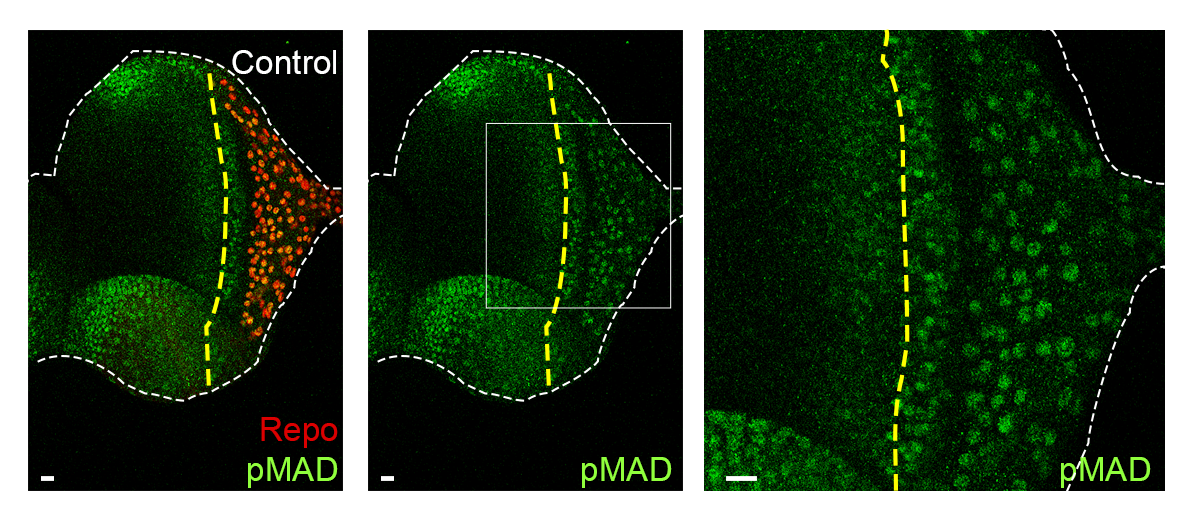

Supplement: S2 Fig — Control eye imaginal discs showing TGF-β activation (pMad). Left panel show glial cells stained with Repo in red. Middle and right panels shows pMad in green. Right panel show a magnification from the inset in the middle panel. A yellow dashed line represents MF. Scale bars correspond to 10 μm. (TIF) [file pgen.1006647.s002.tif]

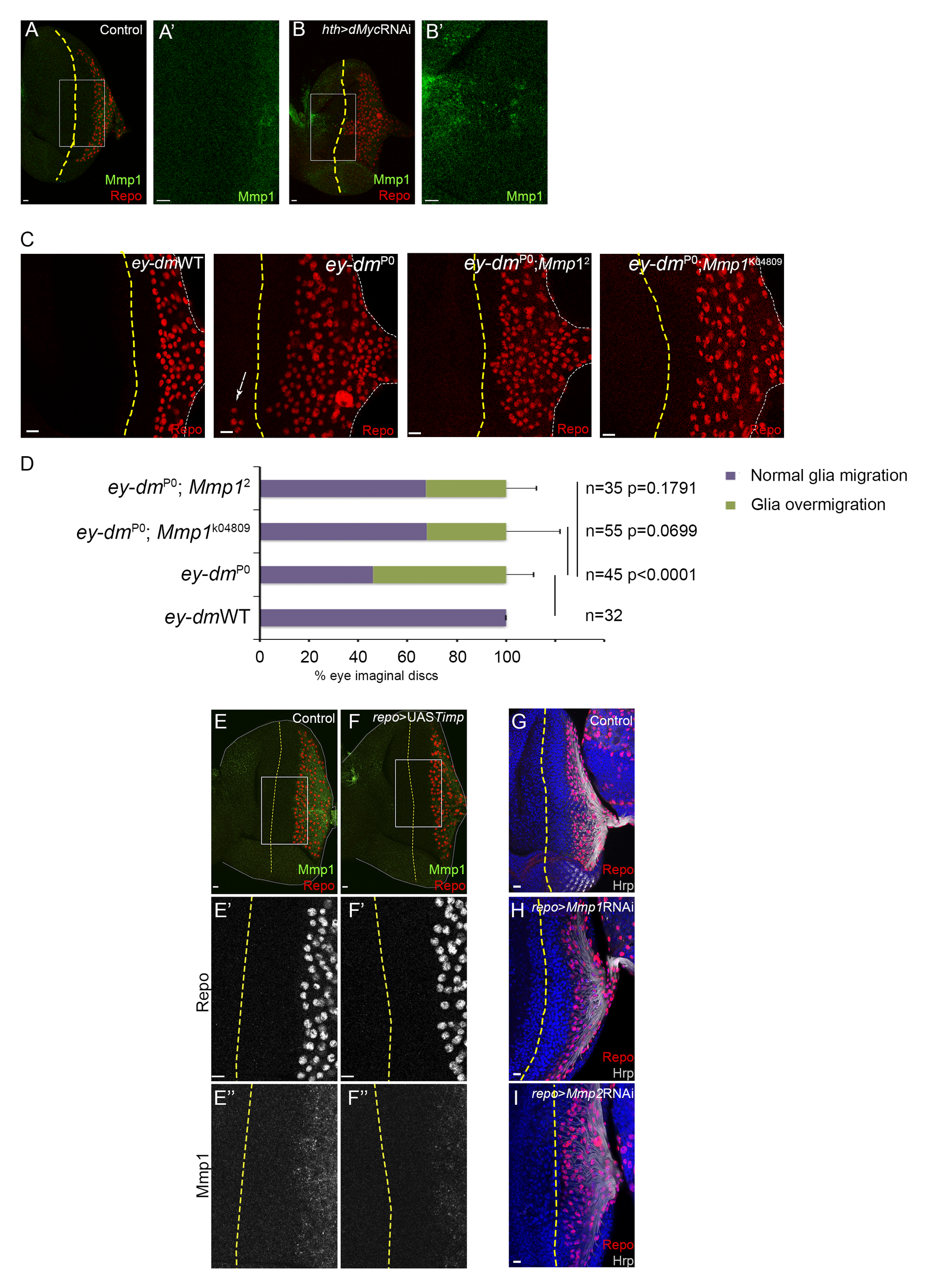

Supplement: S3 Fig — (A and B) Mmp1 expression (green) in control (A) and hth>dMyc RNAi (B). (C) Glia migration in Control (ey-dmWT), dMyc mutant male eye disc (ey-dmP0) and dMyc mutant male eye disc heterozygous for Mmp1 mutant–Mmp12 (ey-dmP0;Mmp12/+) and Mmp1K04809 (ey-dmP0;Mmp1K04809/+). The larvae body (including glia) are dmP0 mutant rescued with dMyc. (D) Graph showing the percentage of eye imaginal discs with glia overmigration vs normal glia migration of the genotypes described on C. (E and F) Mmp1 expression (green) in the Control (E) and repo>UAS-Timp (F). E’ and F’ show Repo magnifications of the inset in E and F while E” and F” show Mmp1 magnification of the same insets. (G–I) When compared with the control (G), downregulation of Mmp1 (H) and Mmp2 (I) in glia (with repo-Gal4) does not interfere with glia migration. Glial cells stained with Repo are shown in red and DAPI stains the nuclei in blue. A yellow dashed line represents the MF. Scale bars correspond to 10 μm. (TIF) [file pgen.1006647.s003.tif]

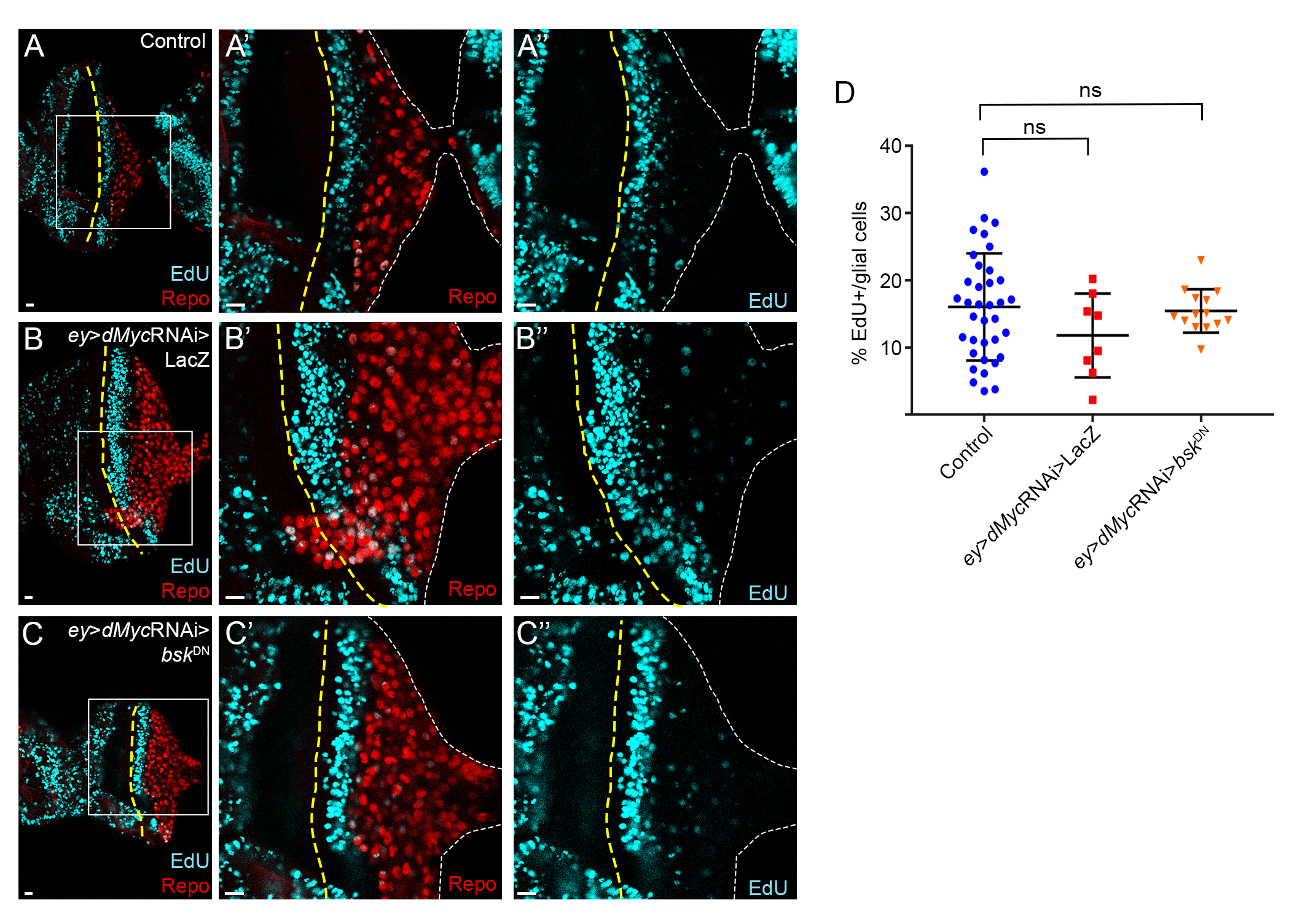

Supplement: S4 Fig — (A–C). EdU staining (light blue) of the control (A), ey>dMyc RNAi>LacZ (B) and ey>dMyc RNAi>bskDN (C). A’–C’ show magnifications of the square inset in A–C. A”–C” show EdU staining magnifications in light blue of the same insets. The region with higher staining of EdU corresponds to the second mitotic wave of photoreceptors differentiation. Glial cells stained with Repo are shown in red; A yellow dashed line represents the MF. Scale bars correspond to 10 μm. (D) Graph showing the percentage of EdU positive glial cells in Control, ey>dMyc RNAi>LacZ and ey>dMyc RNAi>bskDN. (TIF) [file pgen.1006647.s004.tif]

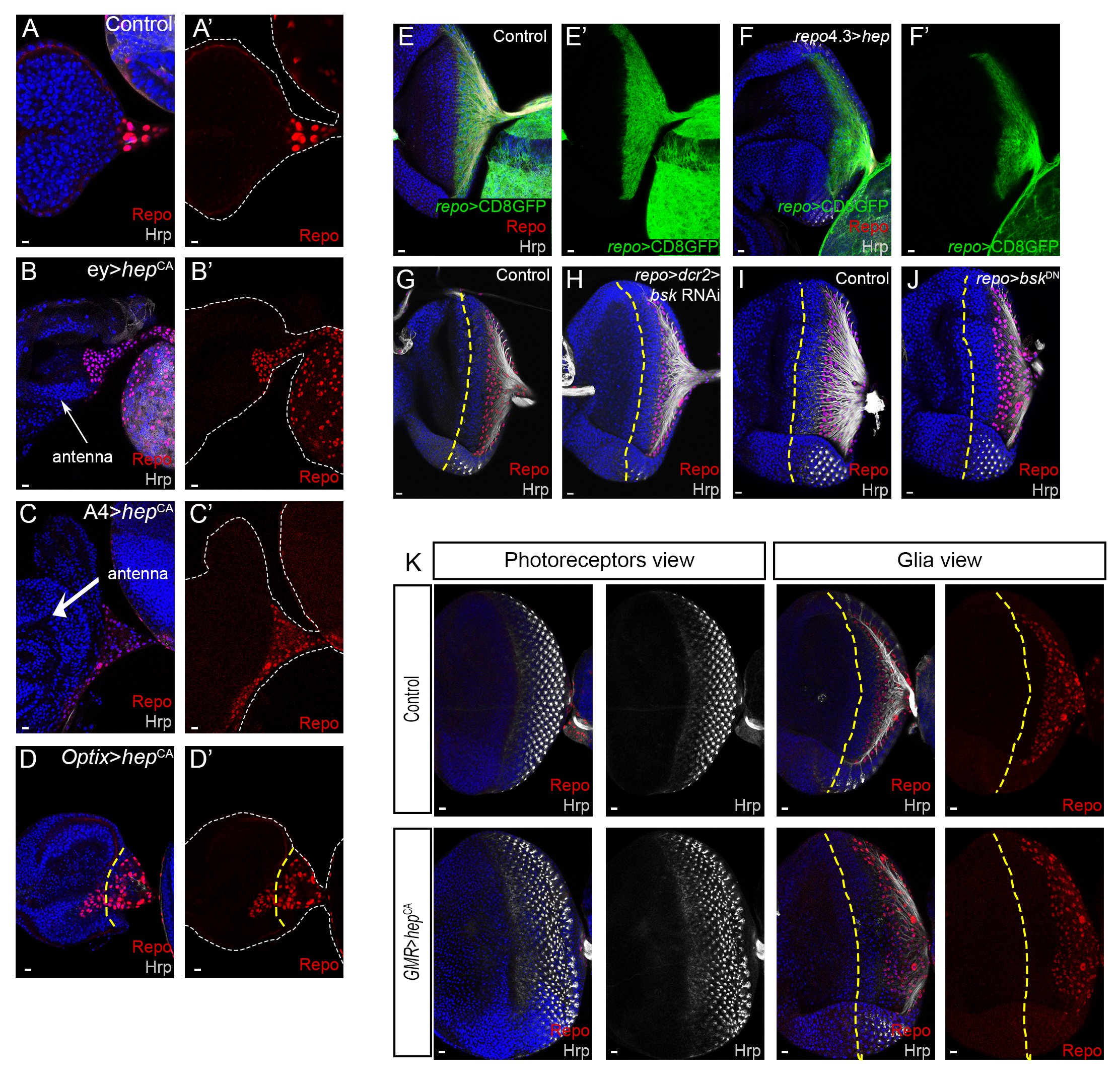

Supplement: S5 Fig — (A–D) Early L3 Control (A) and activation of hep (hepCA) with ey-Gal4 (B), A4-Gal4 (C) and optix-Gal4 (D). (E and F) Control (E) and UAS-hep (F) overexpression in glia (repo4.3-CD8GFP-Gal4). Glial cell membranes are visible in green through the expression of CD8-GFP. (G and H) Control (G) and bsk RNAi in glia (Dcr2;repo-Gal4; H) do not affect glia migration. (I and J) Control (I) and overexpression of bsk dominant negative (bskDN; J) in glia (repo-Gal4) show the same migration pattern of glia as the Control. (K) Photoreceptors and glia view of Control and GMR>hepCA showing normal glia migration. Glial cells stained with Repo are shown in red and DAPI stains the nuclei in blue. Photoreceptors (Hrp) are shown in grey. A yellow dashed line represents the MF. Scale bars correspond to 10 μm. (TIF) [file pgen.1006647.s005.tif]

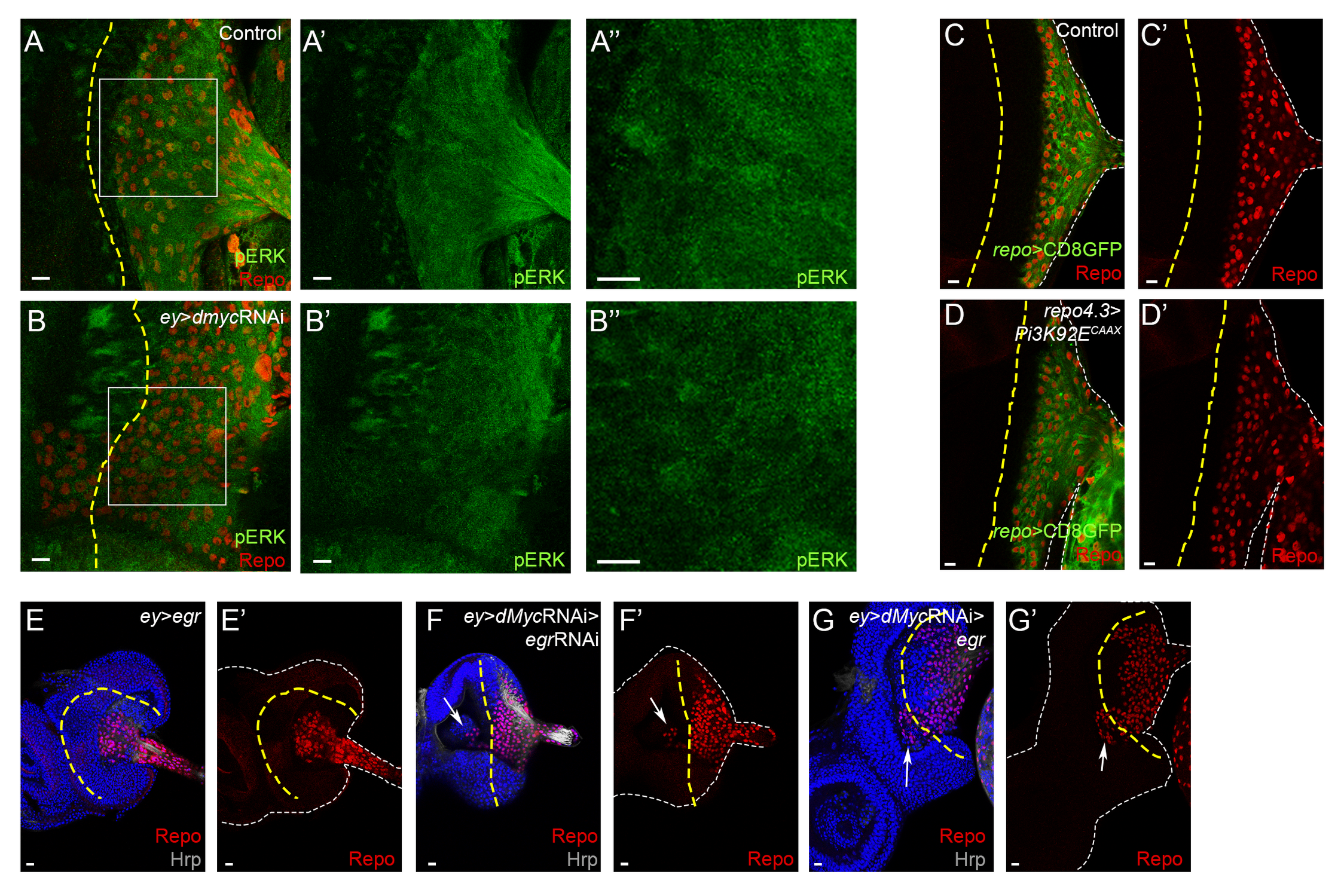

Supplement: S6 Fig — (A and B) pERK staining (green) in Control (A) and ey>dMyc RNAi (B). A’ and B’ show pERK staining and A” and B” show a magnification of the inset represented in A and B respectively. (C and D) Control (C) and Pi3K92E activation in glia using repo4.3-CD8GFP>Pi3K92ECAAX (D). Glial cell membranes are detected in green by CD8GFP expression. (E–G) analysis of Egr role in glia overmigration in ey>egr (E); ey>dMyc RNAi>egr RNAi (F) and ey>dMyc RNAi>egr (G). E’, F’ and G’ show Repo staining. Glial cells are stained with Repo (red), photoreceptors with Hrp (grey) and DAPI counterstains DNA showing the nuclei. A yellow dashed line represents the MF. Scale bars correspond to 10 μm. (TIF) [file pgen.1006647.s006.tif]

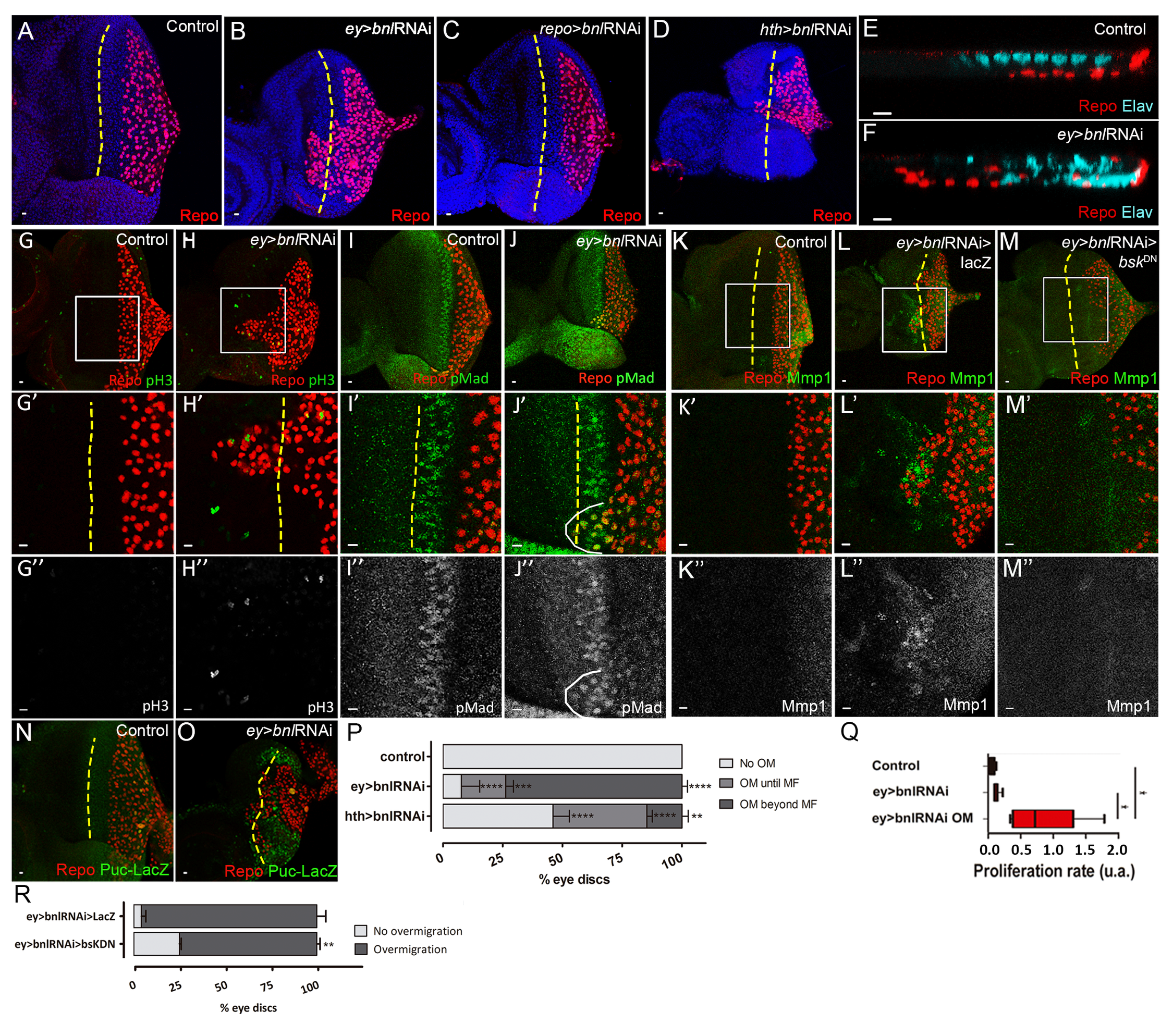

Supplement: S7 Fig — (A–D) Control (A); bnl RNAi in the eye disc with ey-Gal4 (B), in glia with repo-Gal4 (C) and in the anterior domain of the disc with hth-Gal4 (D). (E and F) Transversal analysis of Control (E) and ey>bnl RNAi (F). Photoreceptors are shown by Elav staining in light blue. (G and H) Proliferation analysis of Control (G) and ey>bnl RNAi (H) by pH3 (green). G’, G”, H’ and H” show magnifications of the insets in G and H. (I and J) pMad staining (green) of Control (I), ey>bnl RNAi (J). I’, I”, J’ and J” show magnifications of I and J respectively. (K–M) Mmp1 staining (green) of Control (K), ey>bnl RNAi>LacZ (L) and ey>bnl RNAi >bskDN (M). K’, K”, L’, L”, M’ and M” show magnifications of K, L and M respectively. (N and O) Puc-LacZ expression analysis in pucE69 Control (N) and pucE69; ey>bnl RNAi (O). (P) Percentage of eye discs with glia overmigration in Control, ey>bnl RNAi and hth>bnl RNAi. (Q) Proliferation rate of glia (by pH3) in Control, ey>bnl RNAi and ey>bnl RNAi overmigrating glia (anterior to the MF). (R) Percentage of eye discs with glia overmigration and normal glia migration in ey>bnl RNAi>LacZ and ey>bnl RNAi>bskDN. Glial cells stained with Repo are shown in red and DAPI stains the nuclei in blue. A yellow dashed line represents the MF. Scale bars correspond to 10 μm. (TIF) [file pgen.1006647.s007.tif]

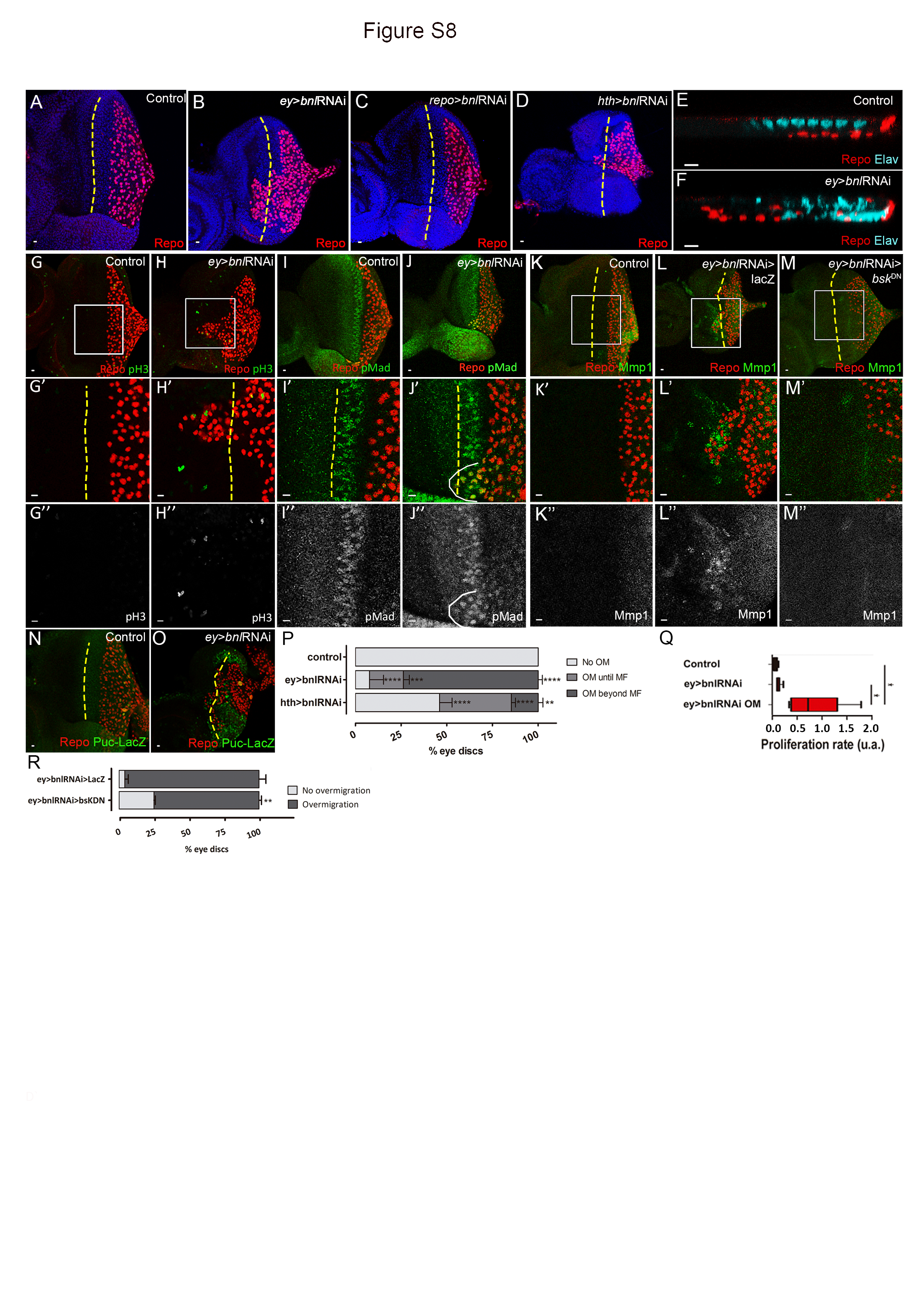

Supplement: S8 Fig — (TIF) [file pgen.1006647.s008.tif]
